# Memory processing by hippocampal adult-born neurons^☆^

**DOI:** 10.1016/j.nlm.2025.108062

**Published:** 2025-05-08

**Authors:** Parimal Chavan, Takashi Kitamura, Masanori Sakaguchi

**Affiliations:** a International Institute for Integrative Sleep Medicine (WPI-IIIS), University of Tsukuba, Tsukuba, Ibaraki 305-8575, Japan; b Institute of Medicine, University of Tsukuba, Tsukuba, Ibaraki 305-0006, Japan; c Department of Psychiatry, University of Texas Southwestern Medical Center, Dallas, TX 75390, USA; d Department of Neuroscience, University of Texas Southwestern Medical Center, Dallas, TX 75390, USA; e Peter O’Donnell Jr. Brain Institute, University of Texas Southwestern Medical Center, Dallas, TX, USA

## Abstract

This review provides an integrative overview of the functional roles of adult neurogenesis in the hippocampal dentate gyrus (DG), focusing specifically on its impact on memory processes across the lifespan. A distinguishing feature of this review is its systematic approach, organizing the contributions of adult-born neurons (ABNs) chronologically through the stages of memory—from initial encoding, through sleep-dependent consolidation, retrieval, and finally forgetting.

Although the existence and extent of adult neurogenesis in the human DG remain debated, accumulating evidence suggests that ABNs support cognitive functions throughout adulthood. This perspective gains particular importance when considering cognitive decline associated with aging and neurological disorders such as Alzheimer’s disease, which are linked to substantial reductions in adult neurogenesis.

We compare traditional models of DG function with emerging evidence highlighting both shared and unique contributions of ABNs. For example, the DG is well-established for its role in pattern separation, and as key mediators of this function, ABNs—due to their transiently heightened plasticity and excitability—appear critical for discriminating novel or similar experiences. On the other hand, recent findings underscore the distinct and essential role of ABNs in memory consolidation during REM sleep, suggesting specialized functions of ABNs that are absent in developmentally born granule cells in the DG.

Clinically, the potential therapeutic importance of enhancing neurogenesis in memory-related disorders, including post-traumatic stress disorder (PTSD), is emphasized, highlighting promising treatments such as memantine. Lastly, we outline key unresolved questions, advocating for future research aimed at understanding ABN-specific mechanisms. Far from being a mere evolutionary vestige, hippocampal ABNs represent dynamic and essential elements of neural plasticity that are critical for memory formation, adaptation, and resilience across the lifespan.

New neurons are generated in the adult brain through a process known as adult neurogenesis, which occurs in the hippocampal dentate gyrus (DG) (Altman and Das, 1965; Eriksson et al., 1998). These adult-born neurons (ABNs) are thought to play a key role in hippocampal circuit dynamics and memory processing (Christian et al., 2014), underpinning the advanced cognitive functions of learning and memory. This review discusses recent advances in understanding the roles of ABNs in memory encoding, consolidation, and retrieval while emphasizing how these cells integrate into pre-existing DG circuits. We further consider how sustaining adult neurogenesis within this spatially constrained niche—and harnessing the regenerative and adaptive capacities of the DG-ABN interplay—may inspire novel therapeutic strategies for treating memory disorders and enhancing brain resilience.

Over the course of vertebrate evolution, adult neurogenesis has appeared to decline as brain complexity has increased. This idea was articulated by Rakic (1985), who argued that advanced brains trade neurogenic plasticity for circuit stability. In other words, once advanced neural circuits underlying higher cognition are established, generating new neurons in adulthood may be unnecessary or even potentially disruptive to finely tuned networks. This hypothesis—later termed the “phylogenetic reduction” of adult neurogenesis—is consistent with the observation that mammals have far fewer neurogenic sites than fish or birds (Lindsey et al., 2006; Kempermann 2012; Kaslin et al., 2008). Thus, the regions where adult neurogenesis persists in more complex brains have been regarded as mere evolutionary remnants that contribute little to higher brain functions.

Challenging this view, Kempermann et al. (2015) proposed that adult neurogenesis in the hippocampal DG may not be a vestige but rather a beneficial and adaptive feature. The DG is rich in synaptic plasticity and deeply involved in memory function (Bliss and Collingridge, 1993; Shimizu et al., 2000; Deng et al., 2010; Goto et al., 2021). Thus, the incorporation of ABNs into this highly plastic circuit may serve to add new computational units to existing networks, potentially enhancing learning and adaptability. This reinterpretation suggests a dynamic evolutionary trajectory in which neurogenesis is preserved—and specialized—exactly where it is needed most.

Comparative studies across vertebrates lend further support to this revised view. For instance, non-mammalian species tend to have lower synaptic densities and simpler neural circuits, yet fish brains contain an abundance of newborn neurons, which are thought to facilitate adaptation to new environments and recovery from injury (Zupanc, 2008). In these simpler synaptic networks, a high level of adult neurogenesis likely functions as a compensatory mechanism.

By contrast, mammalian brains exhibit increased synaptic density and diversity, which supports complex information processing and advanced cognitive function (Moser et al., 1994; Elston et al., 2001; Bartol et al., 2015; Kempermann et al., 2018). For instance, observational fear in mice may underlie emotional empathy by acting as a basis for emotional contagion (e.g., Terranova, 2022). This evolutionary advance may reduce the brain’s reliance on constant neuron replacement. Instead, synaptic plasticity—the strengthening or modification of existing synaptic connections—may play a more important role in learning and memory (Martin et al., 2000; Barnes, 1995). Because of this synaptic complexity, the integration of new neurons may pose a risk of disturbing finely balanced circuits. However, the diversity of neurotransmitters and receptor subtypes observed in mammalian synapses, which also contributes to a highly flexible environment, may in turn provide the necessary conditions for precisely regulated adult neurogenesis to occur. Thus, the integration of ABNs into existing circuits could confer distinct advantages, such as enhanced synaptic plasticity (e.g., Snyder et al., 2001; Saxe et al., 2006) and improved adaptability (e.g., Snyder et al., 2011; Santarelli et al., 2003).

In seeming alignment with the “phylogenetic reduction” model, the existence of adult hippocampal neurogenesis in humans has long been a subject of debate. Whereas some reports suggest that newly formed neurons continue to arise throughout the human lifespan (Eriksson et al., 1998; Spalding et al., 2013; Boldrini et al., 2018), others argue that this process ceases after infancy (Sorrells et al., 2018). However, a comprehensive survey of 57 studies found that 84% support the presence of newborn neurons in the adult human hippocampus (Mendez-David et al., 2022).

Recent investigations also offer detailed molecular insights into newborn neurons in the adult human hippocampus. In particular, by combining single-nucleus RNA sequencing with machine learning, researchers identified immature DG granule cells spanning infancy through old age, revealing that these cells possess unique transcriptional profiles related to neurogenesis and plasticity (Zhou et al., 2022). Thus, although the extent of adult neurogenesis in the human hippocampus remains a topic of ongoing discussion, advanced molecular biology approaches yield compelling evidence that newly generated DG granule cells persist in adulthood and may contribute to hippocampal function and plasticity.

Considering this evolving perspective, a question naturally arises: why does adult neurogenesis persist in humans, who are often regarded as possessing the most complex brains (Gonçalves et al., 2016)? Indeed, studies in rodents show that ABNs play key roles not only in memory-related functions such as pattern separation—the discrimination among similar experiences or cues (Sahay et al., 2011; Clelland et al., 2009)—but also in the regulation of mood and stress resistance (Snyder et al., 2011; Anacker et al., 2018; Santarelli et al., 2003). As far as can be inferred from these rodent studies, adult neurogenesis in humans may be essential for navigating the complexities of our cognitive and emotional worlds.

ABNs undergo a continuous developmental process during which they remain highly plastic compared with fully mature neurons and continue to be modified by ongoing experiences (Christian et al., 2014), which may enable them to play an outsized role in reshaping hippocampal networks. Moreover, recent research shows that ABNs are critical for memory consolidation during sleep (Kumar et al., 2020). This function appears to be unique to ABNs and is not observed in DG granule cells generated during the embryonic period.

In this review, we first elucidate how interactions between the DG and ABNs drive learning and memory, and, based on these insights, illustrate how adult neurogenesis can be harnessed in therapeutic intervention strategies, with examples focusing on PTSD. We hope that this work will also help to survey the evidence for the hypothesis that adult neurogenesis is not merely an “evolutionary vestige” but functions as a dynamic element of neural plasticity, enabling organisms—including mammals—to flexibly adapt to ever-changing environments.

## 1. Circuit integration and unique properties of ABNs

**Fig. 1.**
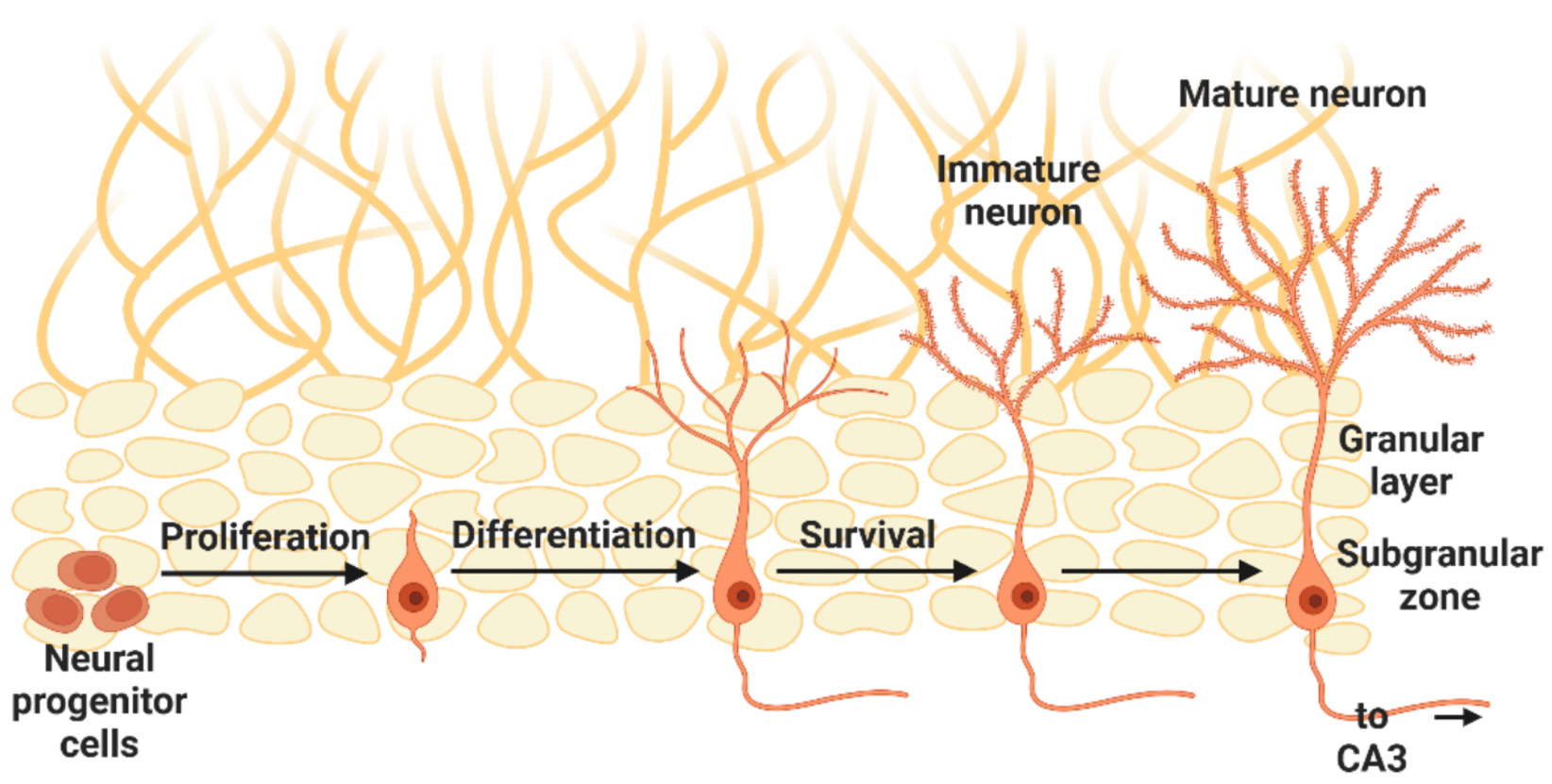
Adult neurogenesis in the DG. Neural progenitor cells in the subgranular zone undergo a series of developmental stages including proliferation, differentiation, and maturation before forming fully mature granule cells. Newly generated neurons that are 4 to 6 weeks old exhibit heightened excitability and synaptic plasticity, which may confer them with unique functions in memory processing not possessed by older neurons.

Adult neurogenesis in the DG is a continuous process that persists throughout life, reflecting a remarkable form of structural plasticity (Ming and Song, 2005; Christian et al., 2014). In rodents, newly generated granule cells in the subgranular zone of the DG can eventually represent a substantial fraction of the overall granule cell population, especially in older animals (Cole et al., 2020). This process begins with the proliferation of radial glia-like neural stem cells (type 1 cells) that give rise to intermediate progenitor cells (type 2 cells) and then to fate-committed neuroblasts (type 3 cells) (Fig. 1). A large proportion of these newly born neurons undergo apoptosis, underscoring the highly regulated nature of the adult neurogenesis process (Tashiro et al. 2006, Sierra et al., 2010). Those that survive proceed through a critical period of maturation, extending dendrites and axons to incorporate into existing hippocampal circuitry (Zhao et al., 2006). Throughout this protracted developmental timeline, both intrinsic molecular programs and extrinsic factors (e.g., exercise, environmental enrichment) modulate cell survival and circuit integration (Christian et al., 2014).

A key feature of ABNs is that they undergo a transient period of heightened excitability and synaptic plasticity approximately 4 to 6 weeks after cell division in adulthood (Schmidt-Hieber et al., 2004; Ge et al., 2007; Gu et al., 2011). During this “young” stage, ABNs display reduced GABAergic inhibition, increased intrinsic excitability, and a lower threshold for long-term potentiation, making them highly responsive to afferent inputs (Ge et al., 2008, Marín-Burgin et al., 2012). The finite duration of this high-plasticity window may allow the hippocampus to integrate new information without destabilization of pre-existing networks (Aimone et al., 2011). In other words, having a population of neurons that transiently exhibit enhanced synaptic plasticity may provide a mechanism for balancing ongoing learning needs with the stability of long-term memories.

**Fig. 2.**
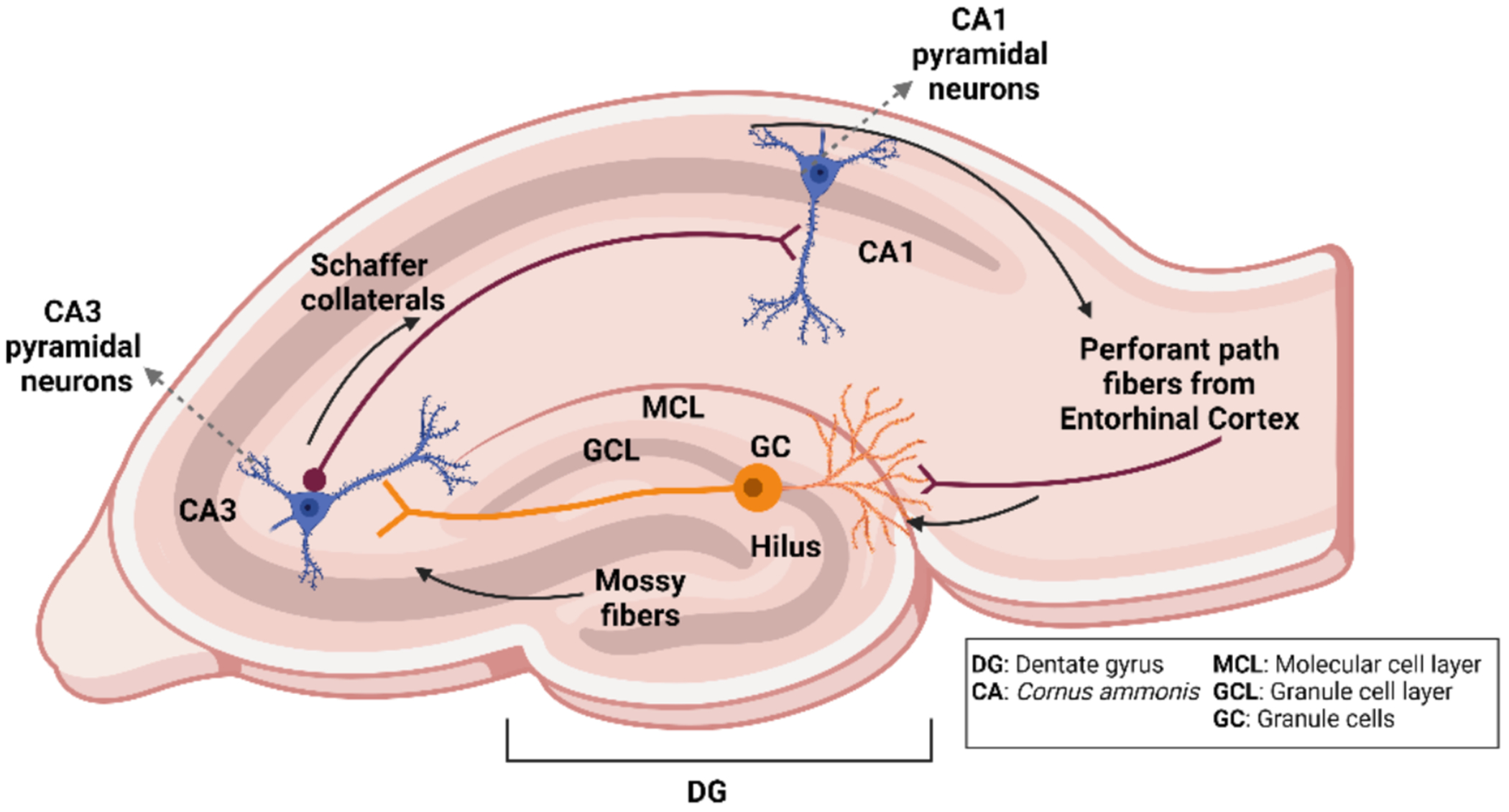
Hippocampal circuitry. Granule cells (GCs) in the DG receive input from the entorhinal cortex via perforant path fibers. The axons of GCs, termed mossy fibers, project to CA3 pyramidal neurons. Information is transmitted from the CA3 to CA1 pyramidal neurons through the Schaffer collateral fibers. MCL, molecular cell layer; GCL, granule cell layer.

As ABNs mature, they receive afferent synapses from local hilar mossy cells and interneurons and later from the entorhinal cortex (Deshpande et al., 2013; Vivar et al., 2012) (Fig. 2). Notably, young ABNs show broad tuning and low input specificity, allowing them to integrate a range of activity patterns (Marín-Burgin et al., 2012, Schmidt-Hieber et al., 2004, Mongiat et al., 2009). Moreover, ABNs receive transient synaptic input from mature granule cells that eventually disappears, further highlighting their evolving role in hippocampal microcircuits (Vivar et al., 2013). By contrast, developmentally born granule cells have higher thresholds for activation, making them sparser encoders (Diamantaki et al., 2016).

Within approximately 3 weeks of cell division, ABNs begin to extend mossy fiber axons into the CA3 and form synapses with pyramidal cells and local interneurons (Fig. 2)—a process resembling the early development of granule cells (Zhao et al., 2006; Hastings and Gould, 1999; Toni et al., 2008; Vivar et al., 2013). Initially, these immature neurons mainly contact local inhibitory interneurons, thus contributing to feedforward inhibition (Restivo et al., 2015; Drew et al., 2016). By around 4 weeks of age, they begin to form substantial excitatory synapses with CA3 pyramidal neurons, aiding hippocampal output (Toni et al., 2008; Faulkner et al., 2008; Gu et al., 2012). This timed progression from local inhibitory circuits to distal excitatory targets helps maintain network stability, as a relatively small number of hyperexcitable ABNs could otherwise destabilize the DG-CA3 circuit (Lacefield et al., 2012). Intriguingly, ABN maturation continues well beyond this early critical phase, with further morphological refinements and increased connectivity to inhibitory interneurons occurring up to 24 weeks of age (Cole et al., 2020).

Recent evidence suggests that young ABNs directly excite mature granule cells in response to medial entorhinal cortex (MEC) input, whereas they exert monosynaptic inhibition of mature granule cells in response to lateral entorhinal cortex (LEC) inputs (Luna et al., 2019). The MEC encodes a global spatial framework, addressing questions like “where am I?” by providing holistic spatial context through path integration mechanisms. By contrast, the LEC processes local cues, addressing questions such as “what is out there?” and “where is it located?” within the environment (Knierim et al., 2013). The differential connectivity of ABNs may contribute to the orthogonalization of LEC- and MEC-derived signals in the DG (Luna et al., 2019).

At 4 to 7 weeks of age, ABNs influence the entire hippocampal network, extending beyond the DG circuit. During this period, the activity of ABNs suppresses spiking activity in the CA3 and CA1 through feedforward inhibition. This likely reduces interference between information, enabling high-dimensional information processing. By contrast, 9- to 12-week-old ABNs are limited to modulating local neuronal activity and do not have a significant impact on network-wide sparsity (McHugh et al., 2022).

The progressive circuit integration of ABNs thus appears to serve multiple functions. Young ABNs may facilitate flexible encoding of new information and pattern separation by virtue of their lower induction threshold and broader responsiveness (Ge et al., 2007, Deng et al., 2010). At the same time, their connection to inhibitory circuitry (Toni et al., 2008; Faulkner et al., 2008) provides a means for regulating higher-dimensional network activity and preventing runaway excitability (McHugh et al., 2022; Lacefield et al., 2012). Over time, as they adopt more mature properties, ABNs become functionally indistinguishable from older granule cells (Laplagne et al., 2006), although subtle differences in connectivity or plasticity may persist (Cole et al., 2020). Ultimately, rather than simply replacing dying cells, adult neurogenesis appears to endow the DG with a continuous supply of immature neurons that preserve and renew hippocampal plasticity.

## 2. Episodic memory

Among the various forms of human memory, declarative memory is unique in that it can be verbally expressed. It encompasses both semantic knowledge, such as recognizing that apples belong to the category of fruit, and episodic memory, which is the ability to consciously recall personal experiences, including the specific details of what happened, where, and when, along with associated thoughts and emotions (Tulving, 1972; Tulving, 2002). Episodic memory lets us mentally revisit past experiences and is vital for forming a coherent narrative of our lives. Although multiple regions of the hippocampus participate in episodic memory, the DG is often highlighted (Hainmueller and Bartos, 2020). In particular, accumulating evidence suggests that ABNs in DG are crucial for the entire process of episodic memory, including its encoding, consolidation, and retrieval, as supported by evidence discussed later in this review.

### 2.1. Engrams, place cells, and cue cells

The concept of memory as a lasting physical trace in the brain dates back to antiquity but was scientifically articulated with the term “engram” in the early 20^th^ century by Semon (1904, 1921), who proposed that experiences modify specific neuron populations that are later reactivated during recall. More recently, Josselyn and Tonegawa (2020) described an engram as a persistent physical and/or chemical alteration in the brain induced by learning that forms the foundation for associations underlying episodic memory. Engram cells are specific populations of neurons that (1) are activated during a learning experience, (2) undergo physical or chemical modifications in response to that experience, and (3) can be reactivated by subsequent exposure to stimuli present during the original learning event, thereby triggering memory retrieval.

In the DG, an example of such engram cells can be seen in “place cells,” which show elevated firing when an animal occupies a particular location, thus forming a cognitive map of the environment (O’Keefe and Dostrovsky, 1971; O’Keefe, 1976; Robinson et al., 2020). DG granule cells exhibit higher spatial selectivity and smaller place fields than CA3 pyramidal cells (Jung and McNaughton, 1993), contributing to the DG’s ability to form finely tuned memory traces. When exploring larger environments, DG neurons often show multiple, scattered place fields (Park et al., 2011), suggesting flexible encoding in more complex contexts.

In tasks in which rewards are located at specific places, DG neurons selectively increase their activity at reward locations (Sasaki et al., 2018). Furthermore, they induce 20-50 Hz resonant activity between the DG and CA3 during the early phase of reward consumption and promote the generation of CA3 network activity (i.e., sharp-wave ripples) after reward consumption via mossy fibers. These activities serve as the foundation for forming neural representations necessary for future behavioral planning (Ylinen et al., 1995; Buzsáki, 2015). In addition, the DG has “cue cells” that respond not to specific locations but to discrete sensory signals such as odors, sounds, or visual patterns, which can incorporate non-spatial information into the engram (Tuncdemir et al., 2022). However, there is still no consensus on whether ABNs possess the properties of engrams.

### 2.2. ABNs and fear memory engrams

Previous studies report that ABNs are activated alongside other DG neurons during the encoding and retrieval of contextual fear memory. Interestingly, however, the ABN ensemble recruited during learning does not appear to be maintained as a lasting component of the memory engram (Kumar et al., 2020; Vergara et al., 2021). Calcium imaging analysis demonstrates that the ABN population activated by fear learning does not persist in its original activity pattern; rather, its firing patterns undergo rapid transformation during the late phase of memory consolidation. During this later phase, a subpopulation of ABNs distinct from those recruited during learning becomes activated (i.e., remapping), and these remapped cells are predominantly active during memory recall (Vergara et al., 2021).

In other words, it is rare for ABNs that were active during learning to be reactivated as engram cells during recall, rendering them a minority within the ABN population engaged at that time. This observation suggests that ABNs do not conform to the conventional definition of engram cells, which posits that the neurons involved in an experience are reactivated during both learning and recall. Nonetheless, it cannot be definitively concluded that the ABNs active during learning are entirely uninvolved in memory retention. To date, no study has selectively targeted the specific subset of ABNs activated during learning to assess their causal impact on subsequent recall (e.g., via optogenetic manipulation of engram cells). Thus, whether artificial reactivation of learning-activated ABNs evokes fear memory recall, or conversely whether their selective inactivation impairs recall, remains untested.

Intriguingly, whereas inhibiting only a small subset of ABNs leads to pronounced deficits in recall (Gu et al., 2012; Park et al., 2016), broader manipulations of the entire DG often yield only modest effects on recall (Bernier et al., 2017; Kheirbek et al., 2013). This discrepancy suggests that ABNs serve a “hub”-like role in regulating the overall representational scheme of the DG network, such as controlling sparsity and pattern separation, via the recruitment of inhibitory circuits. In other words, even though ABNs are few in number, by modulating network dynamics they can influence both the formation and recall of memory engrams (e.g., McHugh et al., 2022).

Moreover, due to their high plasticity, ABNs may be transiently incorporated into memory circuits during the consolidation period, thereby assisting the establishment of memory traces by mature DG neurons. In addition, through their own activity remapping, ABNs may segregate memories of temporally proximate events, contributing to a reduction in memory interference (Rangel et al., 2014; Vergara et al., 2021). Ultimately, as memories become stabilized long-term, ABNs may recede from the core of the engram, becoming less prominent during recall but functioning to reinforce the engram through synaptic modifications at downstream targets. Testing and validating these hypotheses will require further research employing techniques that allow for individual labeling and manipulation of ABNs activated during learning. Current evidence thus supports a view of ABNs as “cells that support engram formation” rather than as principal constituents of the memory engram.

### 2.3. Novelty detection

Novelty detection is a crucial initial step in the memory formation process. Novelty can be broadly categorized into three types. The first type, stimulus novelty, occurs when a stimulus appears that has never been previously experienced (e.g., a novel object or image). The second type, associative novelty, arises when familiar elements are presented in a new arrangement (e.g., a known object appearing in a new location or sequence). The third type, contextual novelty, refers to an unexpected event within a given context (e.g., a red triangle appearing among a series of green triangles) (Kumaran and Maguire, 2007a).

Among these types, associative novelty is closely linked to the hippocampus, as it requires the comparison of new sensory inputs with previously stored memory representations (Kumaran and Maguire, 2007b). In particular, the DG plays a key role in detecting spatial novelty—a subtype of associative novelty characterized by familiar objects placed in new spatial contexts (Hunsaker et al., 2008). Recent findings further indicate that ventral DG mossy cells projecting to dorsal DG granule cells are important for spatial novelty detection (Fredes et al., 2021).

A well-known mechanism of hippocampal novelty detection is Lisman’s “comparator” model, in which the CA1 compares “predictions” from the CA3 with cortical sensory input, generating a novelty signal upon mismatch (Lisman and Otmakhova, 2001). Meanwhile, the CA3 can rapidly learn novel information after only a single trial (Nakazawa et al., 2004; Kitanishi et al., 2015), which in turn influences downstream circuits, including the CA1. However, Chen et al. propose that the supramammillary nucleus (SuM) first routes a coarse contextual novelty signal to the DG (Chen et al., 2020). This suggests a new perspective whereby the SuM detects novelty prior to the hippocampus and acts as an entry point to activate hippocampal networks. Thus, novelty signals may initially arise in the SuM, undergo more detailed processing in the DG, and ultimately coordinate with other hippocampal subfields (CA3-CA1) to facilitate memory encoding and consolidation. In this framework, the DG functions as a critical “gatekeeper,” integrating upstream novelty inputs and orchestrating hippocampal activity to form lasting memories. This suggests that novelty signals from the SuM converge in the DG and initiate hippocampal network activation, potentially enhancing the recruitment of ABNs (Chen et al., 2020; Kirschen et al., 2017; Li et al., 2022). ABNs may thus be preferentially engaged during novel experiences due to their heightened plasticity and excitability (Marín-Burgin et al., 2012), contributing to a robust novelty detection mechanism.

Indeed, mice with specific ablation of young ABNs exhibit increased exploration of novel objects (Denny et al., 2012). Moreover, inhibiting young ABNs through temporally precise optogenetic manipulation impairs the ability to recognize novel objects, whereas activating young ABNs enhances novel object discrimination (McHugh et al., 2022). These pieces of evidence suggest that ABNs play a key role in novelty processing. However, exactly which aspects of novelty detection require ABNs and how they act in concert with DG granule cells to detect novelty remain unclear.

### 2.4. Pattern separation in the DG

Pattern separation, a critical function of the DG, is essential for discriminating between similar memories (Gilbert et al., 1998; Gilbert et al., 2001; Leutgeb et al., 2007; McHugh et al., 2007). Pattern separation enables the discrimination of similar inputs through their transformation into distinct neural representations. This process involves two central mechanisms. First, spatial or global remapping encodes different memories by activating separate populations of neurons with distinct and independent firing locations. Second, rate remapping allows memory encoding by a fixed set of neurons that change their firing rates within their respective firing fields (Leutgeb et al., 2007, McHugh et al., 2007).

In the DG, small changes in input lead to a strong decorrelation of activity among granule cells, resulting in sparse and selective firing patterns (Neunuebel and Knierim, 2012; Neunuebel and Knierim, 2014). This is in contrast to the CA3 region, where different neuron populations are recruited when input differences become larger (Leutgeb et al., 2007). Interestingly, studies show that DG-dependent memory discrimination is not always reliant on place cell remapping. For instance, mice can distinguish between similar contexts without significant changes in place cell firing patterns (van Dijk and Fenton, 2018), indicating the presence of mechanisms beyond spatial or rate remapping. In fact, the DG contains functionally distinct neuronal ensembles defined by Fos or Npas4 expression, each playing opposing roles in context discrimination (Sun et al., 2020). The Npas4-dependent ensemble is critical for promoting contextual fear memory discrimination by receiving enhanced inhibitory inputs from CCK+ interneurons, while the Fos-dependent ensemble—associated with excitatory inputs from the MEC—facilitates generalization.

In addition to these mechanisms, the regulation of downstream hippocampal networks also plays a critical role in shaping memory. The mossy fiber terminals originating from DG granule cells connect to PV+ interneurons in the CA3 and regulate the activity of CA3 pyramidal cells through feedforward inhibition, which enhances memory precision (Ruediger et al., 2011; Guo et al., 2018). This underscores the DG’s essential contribution to the fine-tuned balance between memory discrimination and generalization (Sun et al., 2020).

As another functionally distinct neuronal subtypes in the DG, ABNs play a crucial role in pattern separation. Studies show that increasing adult neurogenesis enhances pattern separation (Sahay et al., 2011), whereas suppressing it impairs fine discrimination abilities (Clelland et al., 2009). However, Huckleberry et al. show that inhibiting ABN activity during memory recall improves context discrimination (Huckleberry et al., 2018). The authors argue that these apparent differences stem from the timing of the intervention—during neurogenesis, learning, or recall. Whereas earlier studies targeted the entire neurogenic process before learning, Huckleberry et al. manipulated ABNs specifically in the recall phase, suggesting that ABNs promote generalization during learning but hinder discrimination during recall. These findings demonstrate that accurately understanding the functions of ABNs requires interventions at the specific phase when the function is expressed, explaining seemingly conflicting results among studies.

Notably, Rangel et al. (2014) report that the DG separates not only spatially overlapping inputs but also temporally distant events. They identified a cell population in the DG that exhibits selective firing for single contexts experienced with a long time gap between exposures—an effect that diminishes when the time gap is shorter or when adult neurogenesis is reduced. This suggests that mature granule cells provide stable and precise representations necessary for memory encoding, whereas the heightened excitability and plasticity of ABNs supply the flexibility to discriminate between input patterns that are similar in space and time.

### 2.5. Memory encoding

The DG plays a crucial role in contextual fear memory encoding (Lee and Kesner, 2004; Kheirbek et al., 2013; Bernier et al., 2017; Hernández-Rabaza et al., 2008). Reactivating DG granule cells that were active during contextual fear learning (via cfos-tag) induces a fear response, demonstrating that these neurons encode contextual fear memory engrams (Liu et al., 2012). Conversely, optogenetic inhibition of these neurons during retrieval (via Arc-tag) impairs memory recall, indicating that learning-activated granule cells are necessary for encoding contextual fear memory (Denny et al., 2014). Neuronal allocation to memory engrams in the DG is influenced by excitability, with CREB positively biasing recruitment (Park et al., 2016). Somatostatinexpressing interneurons in the DG modulate the size of the contextual fear memory engram —in terms of the number of neurons involved—through lateral inhibition (Stefanelli et al., 2016). Somatostatinexpressing interneurons also directly suppress ABN activity (Remmers et al., 2020). In fact, ABNs play critical roles in memory encoding. Mice lacking adult neurogenesis show impaired contextual fear conditioning (Saxe et al., 2006). Also, silencing ABNs during the encoding of a contextual fear memory impairs its acquisition (Danielson et al., 2016; Huckleberry et al., 2018). However, it remains uncertain whether ABNs integrate into engrams as fundamental units of long-term memory or function primarily as modulatory elements within a limited timeframe.

### 2.6. Sleep and memory consolidation

**Fig. 3.**
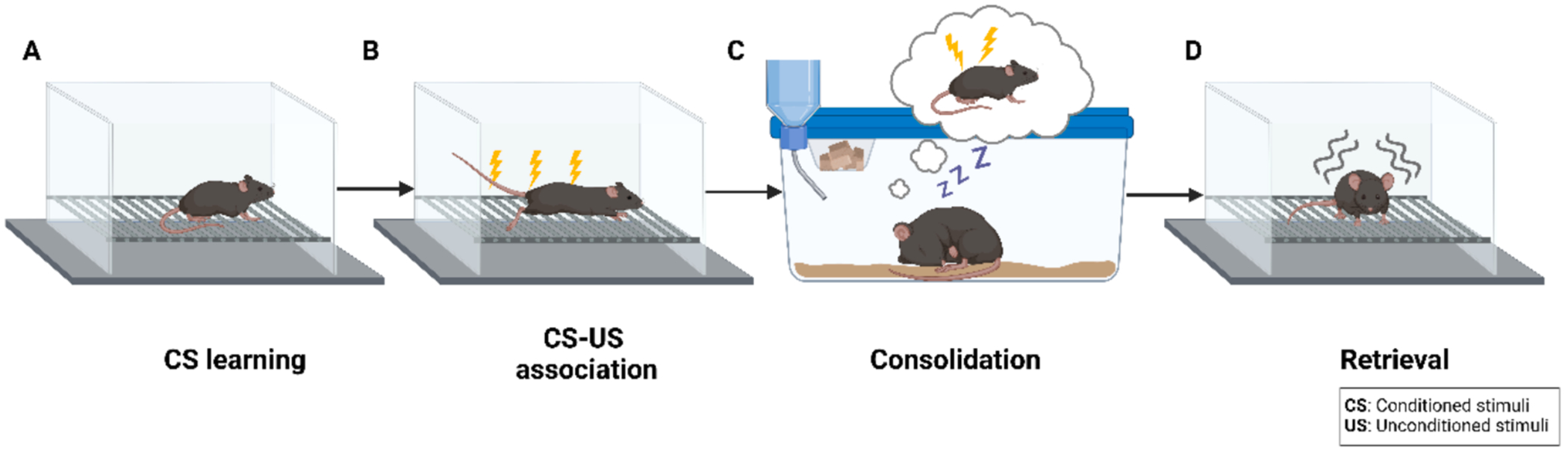
Stages of contextual fear memory processing using a fear conditioning paradigm in rodents. A) Conditioned stimulus (CS) learning in which a mouse is placed in a novel environment (i.e., context). B) CS-unconditioned stimulus (US) association in which the mouse experiences electric foot shock(s) (i.e., the US) while in the context, forming an association between the CS and US. C) The memory of the CS-US association is consolidated, represented by the mouse sleeping and “recalling” its previous experience. D) Retrieval induced by re-exposing the mouse to the context without foot shock, triggering a species-specific fear response (i.e., freezing).

**Fig. 4.**
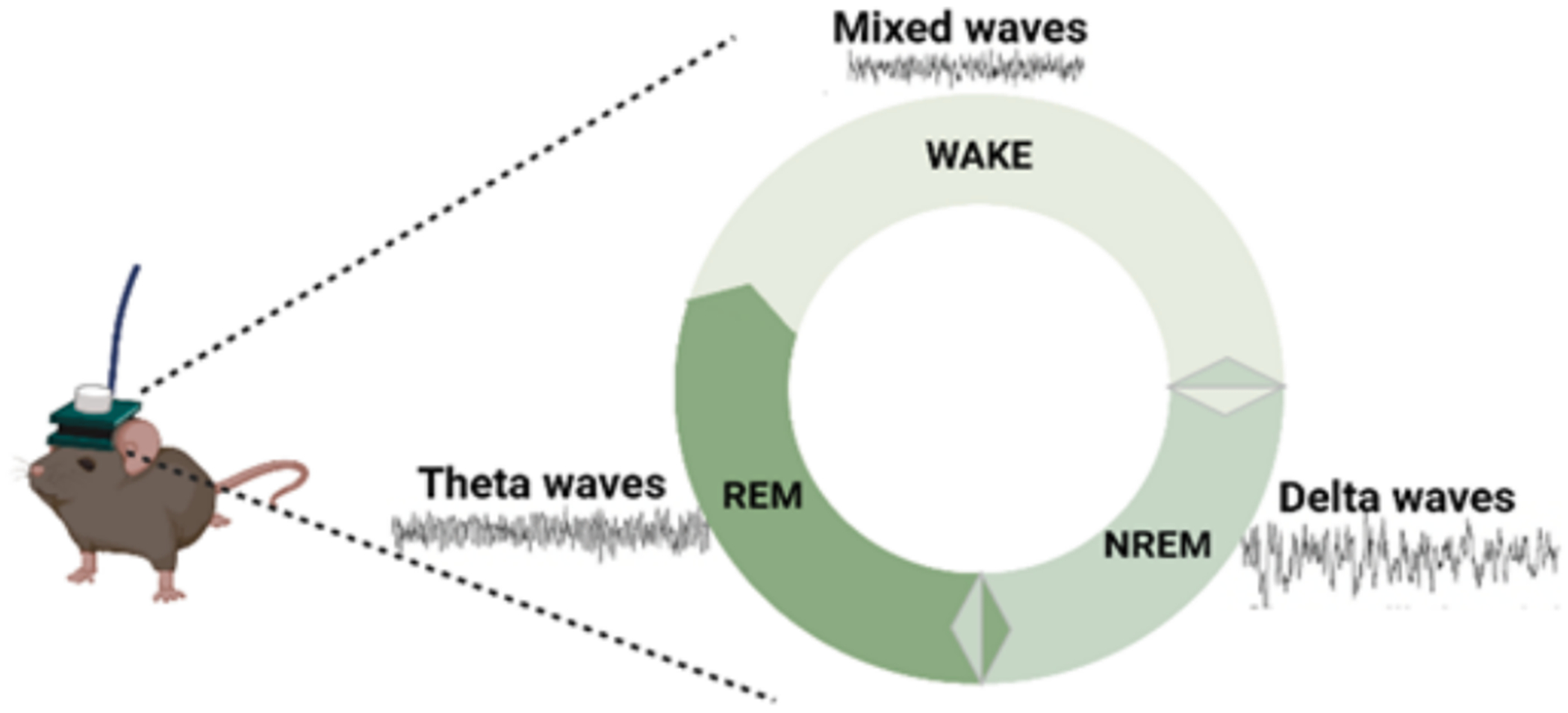
Sleep recording in a mouse showing the three main stages of the sleep cycle with corresponding brain wave patterns: Wake (mixed brain waves, a combination of different frequencies, reflecting the alert or active state of the mouse), NREM (delta waves, which are slow and hig-ampitude), and REM (theta waves, which are lower in amplitude and faster in frequency).

After encoding, memory is consolidated as a long-term memory (Fig. 3). Sleep plays a critical role in this process by facilitating the stabilization and integration of newly acquired information (Diekelmann and Born, 2010). In both humans and rodents, sleep comprises cycles of rapid eye movement (REM) and non-REM (NREM) sleep, each characterized by a distinct pattern of synchronous neural activity across widespread brain regions (Fig. 4).

REM sleep is associated with vivid dreaming (Aserinsky and Kleitman, 1953), with considerable attention focusing on its role in the replay of memory traces (Louie and Wilson, 2001) and the function of such replay in memory processing (Nishida et al., 2009; Sopp et al., 2017), including inference (Abdou et al., 2024). In rodents, REM sleep is marked by prominent synchronized neuronal activity in the theta frequency band (Buzsáki 2002). Inhibiting the generation of these theta oscillations disrupts memory consolidation (Boyce et al., 2016). Hippocampal local oscillations, including theta, are thought to determine the precise timing of neuronal firing and form a critical basis for information processing (Muller et al., 2011). For example, the timing of place cell reactivation at specific phases of the theta oscillation is linked to novelty coding (Poe et al., 2000). Although theta oscillations can be generated independently within each hippocampal subregion, the DG exhibits especially high variability in theta rhythms, presumably due to the diverse inputs it receives (Montgomery et al., 2009; Sullivan et al., 2011). Although activating engram cells in the DG in synchrony with CA1 theta oscillations during wakefulness has been found to enhance memory recall (Rahsepar et al., 2023), the role of engram cell activity synchronized with theta oscillations during REM sleep remains unclear.

**Fig. 5.**
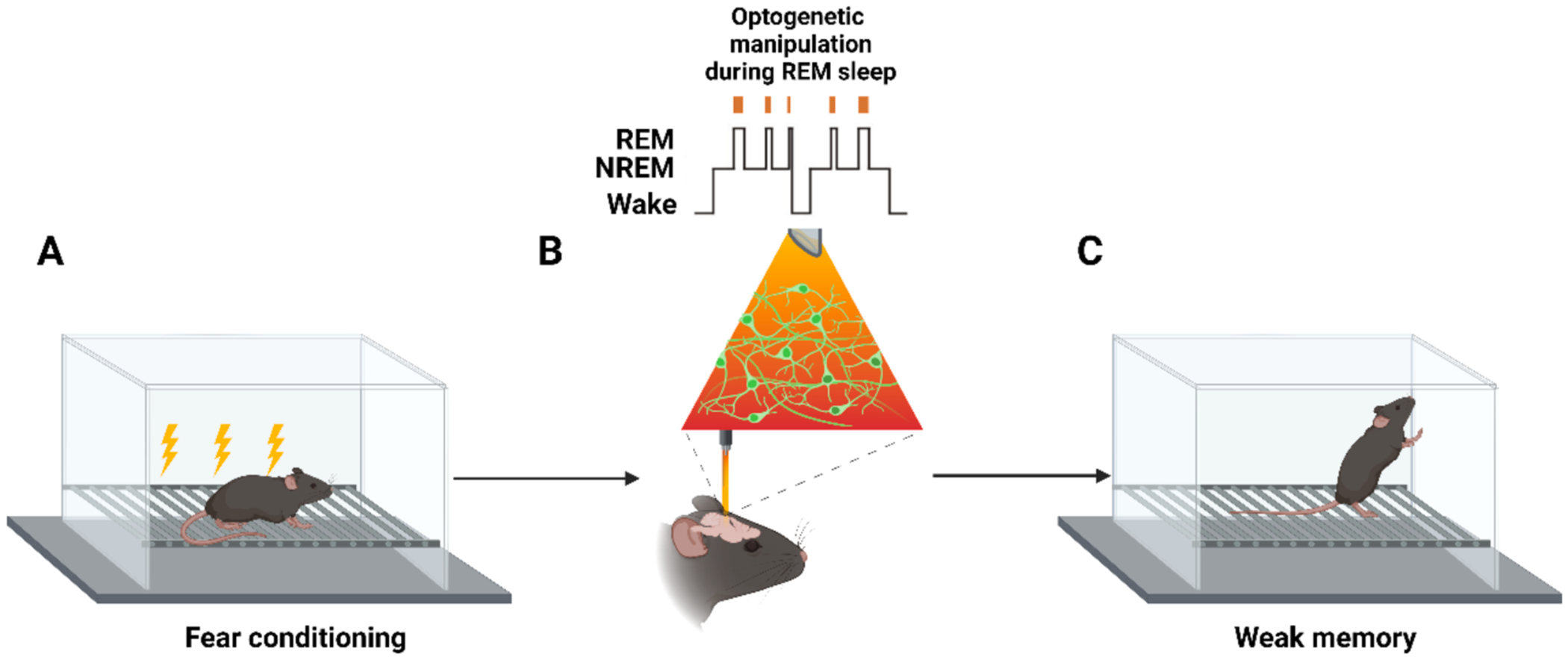
Optogenetic manipulation of ABNs during REM sleep impairs contextual fear memory. A) A mouse undergoes contextual fear conditioning, forming a CS-US association. B) During subsequent REM sleep, ABNs are optogenetically manipulated . C) The mouse exhibits a weaker memory (i.e., reduced freezing) during a memory retrieval test in the same context.

Our previous study (Kumar et al., 2020) identified a subpopulation of ABNs that are reactivated during REM sleep following contextual fear conditioning. Silencing ABNs during REM sleep impairs memory consolidation (Fig. 5), whereas random activation of ABNs during REM sleep disrupts memory consolidation. This suggests that the precise timing and pattern of ABN activity—possibly synchronized with REM-theta oscillations—are critical for memory consolidation.

ABNs also influence the temporal dynamics of hippocampusdependent memory storage. Impaired adult neurogenesis in the DG prolongs the hippocampus dependency of contextual fear memory, while promoting adult neurogenesis accelerates the transition of memory storage from the hippocampus to extra-hippocampal regions (Kitamura et al., 2009). This suggests that ABNs facilitate the consolidation and reorganization of memories over time, contributing to the efficient transfer of information for long-term storage.

### 2.7. Memory retrieval

Previous studies demonstrate the critical role of the DG in memory retrieval (Bernier et al., 2017; Carretero-Guillén et al., 2024; McHugh et al., 2007). In humans, the DG is particularly important for memory recall under conditions of high similarity between stimuli or insufficient retrieval cues (Stevenson et al., 2020). In mice, experimental evidence indicates that reactivation of sparse neuronal ensembles within the DG that were originally recruited during encoding is sufficient to induce memory recall (Liu et al., 2012). Indeed, DG engram neurons regulate context-dependent memory retrieval by reinstating neural states corresponding to specific contexts in downstream regions, including the CA1 (Coelho et., 2024). In upstream regions, glutamate release from the SuM-DG circuit and its high synchrony are essential for the retrieval of novel place recognition memory (Li et al., 2020). The SuM directly projects to ABNs (Li et al., 2022). Indeed, silencing young ABNs—but not other ages of ABNs—during memory retrieval impairs memory recall (Gu et al., 2012; Huckleberry et al., 2018). Stimulation of the SuM increases neurogenesis itself, leading to a significant improvement in memory retrieval (Li et al., 2022). These results suggest that young ABNs work in concert with the SuM for memory retrieval. However, it is still unknown whether the observed behavioral effects stem from the integration of ABNs in the memory engram or from their support of the reactivation of engrams mediated by other neurons.

### 2.8. Forgetting

Forgetting has been conceptualized as a failure to retrieve information that was previously accessible (Tulving, 1974), often due to disruption in memory consolidation or interference during retrieval (Anderson et al., 1994). Forgetting has also been defined as the failure of a retrieval cue to reinvoke the pattern of neural activity present at encoding (Tulving and Thomson, 1973; Polyn et al., 2005; Ryan et al., 2015; Frankland et al., 2013). ABNs in the hippocampus are suggested to contribute to the forgetting of existing memories. Akers et al. (2014) demonstrated that elevated hippocampal neurogenesis promotes forgetting by remodeling existing memory circuits, particularly during infancy. Building on this, Guskjolen et al. (2018) showed that memories lost during infantile forgetting are not erased but can be recovered by optogenetic reactivation of encoding engram cells. Increasing hippocampal neurogenesis leads to the forgetting of recently acquired contextual fear memories but spares older memories, suggesting an agedependent sensitivity of memories to neurogenesis-induced forgetting (Gao et al., 2018). Neurogenesis-induced forgetting of hippocampusdependent memory also occurs in rats across multiple behavioral tasks (Scott et al., 2021).

Mice with depleted neurogenesis showed increased memory interference, suggesting that ABNs reduce forgetting by separating similar memories (Tronel et al., 2012). Further supporting the link between adult neurogenesis and forgetting, Epp et al. (2016) demonstrated that increased neurogenesis after learning impairs memory recall in mice. This suggests that ABNs actively remodel hippocampal circuits to reduce proactive interference, facilitating the encoding of new experiences while destabilizing older memories (Epp et al., 2016; Epp et al., 2021). A recent study also shows the importance of ABNs in forgetting maladaptive memories, as enhanced neurogenesis leads to the weakening of conditioned place preference (Ávila-Gámiz et al., 2025).

Similar to how exercise and the antidepressant fluoxetine increase the number of ABNs, administration of memantine also augments hippocampal neurogenesis and leads to a marked reduction in conditioned fear responses (e.g., freezing), effectively erasing memory (Akers et al., 2014; Ishikawa et al., 2016; Ishikawa et al., 2019). Memantine is a noncompetitive antagonist of the NMDA glutamate receptor that protects neurons from excessive excitatory signaling, thereby mitigating cognitive decline. In the 2000s, memantine was approved in Europe and the United States for moderate-to-severe Alzheimer’s disease, making it the first Alzheimer’s disease drug apart from cholinesterase inhibitors. Interestingly, animal studies report that memantine promotes neurogenesis in the adult hippocampus, which may underlie its facilitation of forgetting (Jin et al., 2006; Maekawa et al., 2009; Cahill et al., 2019).

## 3. PTSD

Normally, when an individual is repeatedly exposed to cues that trigger a fear memory without experiencing the original trauma, the fear response gradually diminishes through a process known as memory extinction (Jones, 1924; Falls et al., 1992; Phelps et al., 2004). In contrast, PTSD is characterized by persistent avoidance of trauma-related cues, preventing extinction from occurring and causing patients to relive the same intense fear response each time the memory is recalled, severely impairing daily life (Ehlers and Clark, 2000; American Psychiatric Association, 2013; Yehuda et al., 2015). An effective treatment for PTSD, called prolonged exposure therapy, involves deliberately and repeatedly recalling the traumatic memory in a safe environment (Hamblen et al., 2019; McLean et al., 2022; Schnurr et al., 2022). This process is thought to leverage the mechanism whereby recalling a memory destabilizes it, allowing extinction to be promoted during its reconsolidation phase (Monfils et al., 2009; Schiller et al., 2010). Ishikawa et al. (2016) demonstrated that administering memantine during this reconsolidation window can attenuate subsequent fear responses.

Building on these basic research findings, Hori et al., (2021) conducted a 12-week open-label trial of memantine in 13 civilian female PTSD patients, adding the drug to existing regimens with minimal changes to other medications. Participants’ self-reported PTSD Diagnostic Scale (PDS) scores fell substantially (mean 32.3 → 12.2; d = 1.35), with notable improvements across re-experiencing, avoidance, and hyperarousal clusters. Six of the ten completers achieved remission, and only mild side effects (insomnia, drowsiness, weight change) were reported. While these results suggest memantine’s potential to alleviate PTSD symptoms, the absence of a placebo control underscores the need for larger randomized trials.

Although memantine-induced increases in neurogenesis appear to promote memory forgetting in mice, it remains unclear whether the same mechanisms operate in humans, in which the number of ABNs is believed to be substantially lower. Additionally, further studies are required to clarify whether the symptom improvement observed in PTSD patients is attributable to ABNs or other NMDA receptor-mediated processes (Monfils et al., 2009).

## 4 Future perspectives

Recent research increasingly shows that ABNs in the DG provide a high level of structural and functional plasticity. As outlined in this review, ABNs differ from developmentally born granule cells not only in their elevated excitability and time-limited plasticity but also in their contributions to each stage of the memory process. Here, we suggest several perspectives that build upon these findings, aiming to address the question of why adult neurogenesis exists.

## 5. Significance of adult neurogenesis in humans

Although the existence and extent of adult neurogenesis in the human hippocampus remain debated, multiple lines of evidence including molecular markers, advanced imaging, and single-cell transcriptomics support the idea that neurogenesis persists—at least to some degree—throughout adulthood. Elucidating whether and to what extent these neurons integrate into existing circuits in humans is a major goal for bridging animal studies and human neuroscience. Considering disorders in which brain functions involving ABNs are impaired (e.g., Alzheimer’s disease) together with detailed single-cell analyses of the developmental trajectories of ABNs is expected to further elucidate the role of adult neurogenesis in humans.

## 6. Integrating ABN plasticity into DG network complexity

The DG processes diverse inputs from spatial to emotional contexts, relying heavily on its inherent plasticity. ABNs, with their transient heightened excitability and plasticity, are proposed to modulate network dynamics in concert with mature neurons. Future research must determine precisely how these two populations coordinate their activity, how inhibitory and excitatory signaling shift under different behavioral states (e.g., learning, stress, sleep), and how these shifts impact longterm information encoding in the DG. Combining *in vivo* circuit monitoring with cell type-specific manipulations will be critical for unraveling these complex interactions.

## 7. Reconstructing memory consolidation models with a focus on sleep

Sleep plays an essential role in transferring newly acquired information from transient to long-term storage. Recent findings suggest that young ABNs re-engage in neural activity during REM sleep to bolster memory consolidation. Future work should examine how newly born and mature neurons in the hippocampus interact during various sleep stages and frequencies (e.g., theta) to stabilize memory traces. This line of inquiry could illuminate both fundamental aspects of sleep physiology and the specialized contributions of ABNs to memory.

## 8. Clinical and applied perspectives

The recognition that ABNs contribute to cognitive functions rather than merely replacing lost neurons has energized new therapeutic ideas. The discovery of molecular and environmental factors regulating ABN survival and integration into the DG may inform strategies for therapeutically enhancing neurogenesis in memory disorders, mood disorders, and neurodegenerative diseases (e.g., Li et al., 2023). In particular, there are high expectations for future research to apply insights gained from adult neurogenesis when using cell sources such as induced pluripotent stem cells to regenerate lost neural circuits. Key areas of interest include the mechanisms by which transplanted cells engraft, integrate into existing circuits, and become functionally active.

By adopting these perspectives, we can move beyond seeing adult neurogenesis as an evolutionary vestige. Instead, the continued study of ABNs—along with their unique developmental timeline and circuitintegration patterns—promises to expand our understanding of the remarkable adaptability of the hippocampus. As multidisciplinary approaches in genetics, systems neuroscience, and computational modeling converge, clarifying the many facets of ABN function will not only deepen our grasp of how the brain encodes, consolidates, and retrieves memories but may also open pathways for innovative treatments for cognitive and affective disorders.

## Data Availability

No data was used for the research described in the article.
